# Treatment algorithm with dexamethasone intravitreal implant in patients with diabetic macular edema

**DOI:** 10.1111/aos.14339

**Published:** 2019-12-29

**Authors:** David Epstein, Pierfrancesco Mirabelli, Monica Lövestam Adrian

**Affiliations:** ^1^ Department of Ophthalmology Sankt Erik Eye Hospital Karolinska Institutet Stockholm Sweden; ^2^ Department of Ophthalmology Linköping University Linköping Sweden; ^3^ Department of Ophthalmology Lund University Lund Sweden

Editor,

Anti‐VEGF (vascular endothelial growth factor) treatment for wet age‐related macular degeneration (AMD) and diabetic macular edema (DME) is well established. Since it requires many clinical visits and frequent injections, different treatment regimens have been discussed, from monthly treatment to pro re nata (treatment when needed) and treat and extend (T&E), a proactive treatment regimen where injection is given at every follow‐up visit with extended intervals. In the treatment of wet AMD, T&E regimen has been shown to be superior to other regimens with a better visual outcome despite fewer injections (Augsburger et al. 2019).

Ozurdex (dexamethasone 0.7 mg implant) is also available for the treatment of DME and has shown comparable results with anti‐VEGF in both gain of Visual Acuity (VA) and reduction of retinal thickness (Boyer et al. 2014). The possible side‐effects of intravitreal dexamethasone implant, that is cataract development and increased intraocular pressure (IOP), have made it a second‐choice drug in the treatment of DME. The European guidelines (Schmidt‐Erfurth et al. 2017) recommended the use of Ozurdex in patients not responding to three to six anti‐VEGF injections. However, Ozurdex could be considered as first‐line treatment in pseudophakic eyes, in patients with anamnesis of a recent major vascular event and in cases with expected poor compliance.

In the clinical trials (Boyer et al. 2014), Ozurdex was administered every 6 months. However, most real‐world data report a maintained effect around 4 months. In clinical practice, the treatment regimen used with Ozurdex is pro re nata. The patient receives an Ozurdex injection, within 1 month IOP is measured, and after 2 months the effect is assessed. The next follow‐up visit is scheduled after another 2 months, that is at month 4 after the injection. If recurrence of edema is detected, the patient is reinjected; otherwise, a new appointment is set after another 2 months. With this approach, the patient is treated only when a recurrence of edema is seen. This may jeopardize retinal structure and function and result in a poor visual outcome over time.

**Figure 1:**
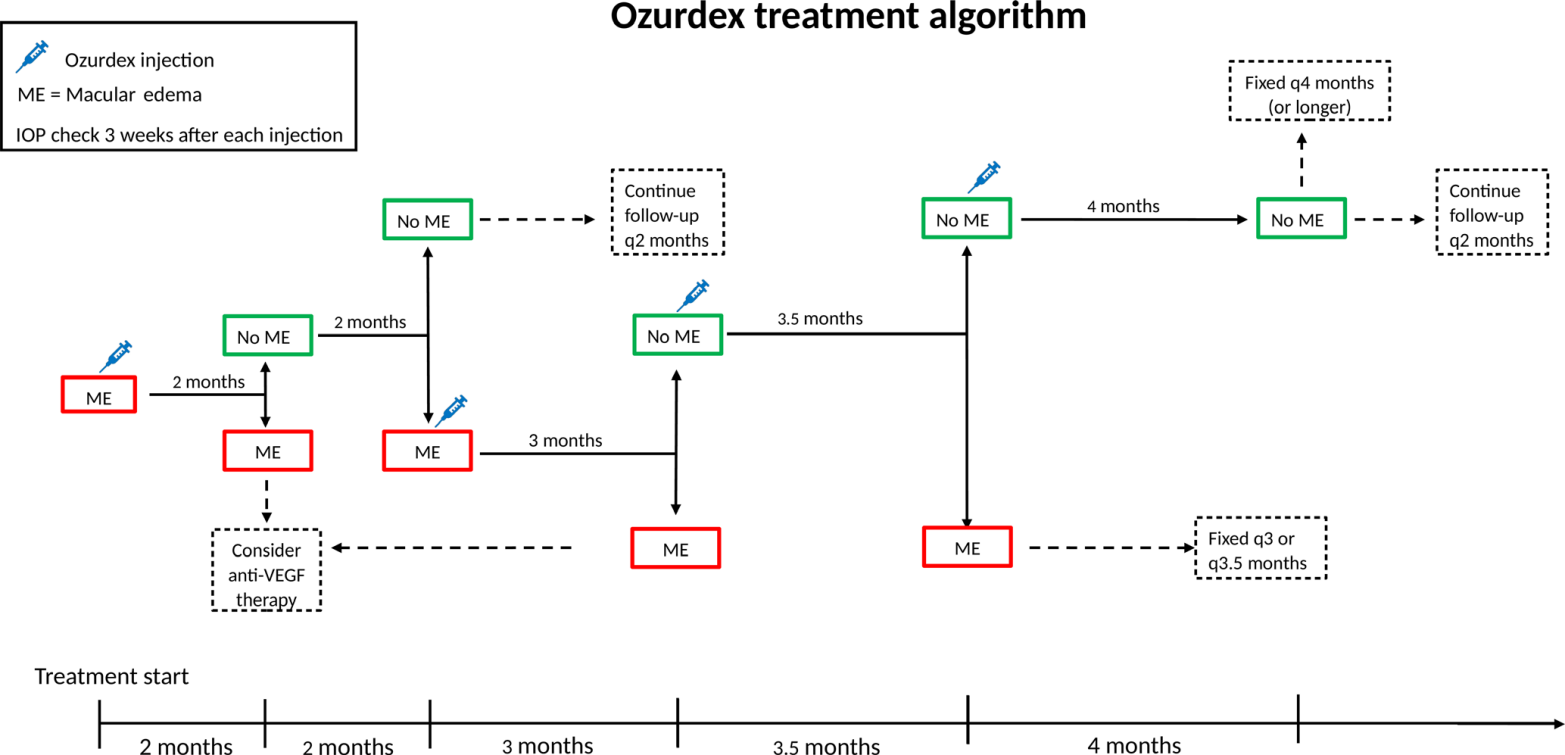
First follow‐up after the injection; month 2. Good responder; next visit month 4; No responder; stop treatment. Recurrence of edema at month 4; reinjection. Next visit; month 3; No recurrence month 4; observation with fixed intervals. Edema at month 4 but no edema at month 3, reinjection on a dry retina. Next visit; 3.5 months. If incipient edema at month 3.5, the patient is scheduled for treatment with fixed intervals between 3 and 3.5 months. No edema at month 3.5; next interval can be extended to 4 months.

In a recent Spanish study, a proactive treatment protocol, describing the time‐point for follow‐up visits and retreatments, has been illustrated (García‐Layana et al. 2018). In a Swedish study group, we have developed a treatment algorithm for dexamethasone, with intent to optimize the number of injections, follow‐up visits and treatment outcome (Fig. 1). The intention is that this algorithm would work as a useful tool for clinicians to optimize and facilitate the treatment with Ozurdex, once the decision for initiating the treatment has been made.
